# Three Amino Acid Substitutions in the Spike Protein Enable the Coronavirus Porcine Epidemic Diarrhea Virus To Infect Vero Cells

**DOI:** 10.1128/spectrum.03872-22

**Published:** 2022-12-13

**Authors:** Bingqing Chen, Shijuan Dong, Li Yu, Fusheng Si, Chunhua Li, Chunfang Xie, Ruisong Yu, Zhen Li

**Affiliations:** a Institute of Animal Husbandry and Veterinary Science, Shanghai Key Laboratory of Agricultural Genetics and Breeding, Shanghai Engineering Research Center of Breeding Pig, Shanghai Academy of Agricultural Sciences, Shanghai, China; Utrecht Institute for Pharmaceutical Sciences, Utrecht University

**Keywords:** PEDV, cell adaptation, S protein, tropism, reverse genetics

## Abstract

Porcine epidemic diarrhea virus (PEDV), a continuously evolving pathogen, causes severe diarrhea in piglets, with high mortality rates. To prevent or mitigate the disease, it is common practice to develop live or inactivated PEDV vaccines based on cell-adapted viral variants. Propagating wild-type PEDV in cultured cells is, however, often challenging due to the lack of knowledge about the requirements for the cell adaptation of PEDV. In the present study, by using the RNA-targeted reverse genetic system for PEDV to apply S protein swapping followed by the rescue of the recombinant viruses, three key amino acid mutations in the S protein, A605E, E633Q, and R891G, were identified, which enable attenuated PEDV strain DR13 (DR13^att^) to efficiently and productively infect Vero cells, in contrast to the parental DR13 strain (DR13^par^). The former two key mutations reside inside and in the vicinity of the receptor binding domain (RBD), respectively, while the latter occurs at the N-terminal end of the fusion peptide (FP). Besides the three key mutations, other mutations in the S protein further enhanced the infection efficiency of the recombinant viruses. We hypothesize that the three mutations changed PEDV tropism by altering the S2′ cleavage site and the RBD structure. This study provides basic molecular insight into cell adaptation by PEDV, which is also relevant for vaccine design.

**IMPORTANCE** Porcine epidemic diarrhea virus (PEDV) is a lethal pathogen for newborn piglets, and an efficient vaccine is needed urgently. However, propagating wild-type PEDV in cultured cells for vaccine development is still challenging due to the lack of knowledge about the mechanism of the cell adaptation of PEDV. In this study, we found that three amino acid mutations, A605E, E633Q, and R891G, in the spike protein of the Vero cell-adapted PEDV strain DR13^att^ were critical for its cell adaptation. After analyzing the mutation sites in the spike protein, we hypothesize that the cell adaptation of DR13^att^ was achieved by altering the S2′ cleavage site and the RBD structure. This study provides new molecular insight into the mechanism of PEDV culture adaptation and new strategies for PEDV vaccine design.

## INTRODUCTION

Porcine epidemic diarrhea virus (PEDV) is a pathogen that leads to a severe enteric disease of pigs, PED (porcine epidemic diarrhea), of which the symptoms include watery diarrhea, vomiting, and anorexia and for which the rate of mortality of infected piglets can reach as high as 100% (1, 2). Outbreaks of PED have brought huge economic losses to the pig industry worldwide since its emergence in the 1970s. Especially since 2010, variants of PEDV that were classified mainly as genotype II emerged and caused severe epidemics of PED in many countries; genomic analyses revealed extensive mutations within the prevalent strains (3, 4). Clinical surveys showed that the vaccines developed from classical genotype I PEDV could not provide efficient protection against the genotype II variants (5–7).

PEDV belongs to the genus *Alphacoronavirus* of the family *Coronaviridae*. It is an enveloped virus with single-stranded positive-sense genomic RNA (8, 9) encoding nonstructural proteins, structural proteins, and one accessory protein, ORF3 (open reading frame 3). The nonstructural proteins are translated in the form of polyprotein 1a (pp1a) and pp1ab, which are preproteins of several enzymes related to virus replication. The structural proteins include the spike (S) protein, the envelope (E) protein, the membrane (M) protein, and the nucleocapsid (N) protein, each of which is an essential component of virus particles. PEDV can replicate efficiently in enterocytes of porcine intestinal villi, causing cell rupture and severe villus atrophy (1, 2).

It is generally difficult to grow wild-type PEDV in cultured cells. The first successful PEDV propagation *in vitro* was reported in 1988 with Vero cells (10), a well-known cell line isolated from African green monkey kidney cells. In this report, a detectable cytopathic effect (CPE), including syncytia with fewer than 10 nuclei inside, was observed after the cells were inoculated with intestinal isolates. However, more extensive syncytia with more than 100 nuclei inside appeared when the inoculum was passaged three more times. This phenomenon indicated that the virus needed adaptation to become more efficient in infecting and replicating in cultured cells. Later on, several PEDV strains were adapted for cell growth in Japan, South Korea, Belgium, and China. For instance, the DR13 strain isolated from South Korea in the 1990s not only propagated in Vero cells efficiently without trypsin but also acquired attenuated pathogenicity after serial passage (11). In fact, the probability of PEDV isolation from morbid tissues in cultured cells is very low, and trypsin is usually indispensable at the beginning of the isolation process (12).

Despite the successful isolation of several cell-adapted PEDV strains, the molecular mechanisms of their adaptation to cells are still unknown. Theoretically, due to their important role in receptor binding and cell entry, S proteins of coronaviruses are the main targets for studying tropism and cell adaption (13). It is also a kind of tropism switching for the viruses to adapt to cultured cells if the cells are not primary or immortalized cells derived from a natural host. There are quite a number of tropism switching studies with different coronaviruses, and these studies were not exceptionally about the genetic manipulation of S proteins. For instance, the substitution of 2 amino acids (aa) at the N terminus of the S protein of TGEV (transmissible gastroenteritis virus) could change the enterocyte tropism of the virus (14). A milestone study with feline infectious peritonitis virus (FIPV) showed that the replacement of the spike protein ectodomain with that from mouse hepatitis virus (MHV) made the recombinant virus switch its tropism to rodent cells (15). An opposite study with MHV found that 21 amino acid substitutions and a 7-amino-acid insertion made its tropism extend to monkey or feline cells, further proving the critical role of the S protein in coronavirus tropism (16). There are several examples of research on the mechanism of PEDV adaptation. A previous study showed that 34 blind passages of the PEDV 83P-5 strain on Vero cells made the virus adapt to the cells and brought about mutations of 6 nucleotides (nt) in the S gene, leading to 5 amino acid substitutions in the signal peptide and the ectodomain of the S protein (17). A recent study revealed that the cellular adaptability of recombinant PEDV (rPEDV) depended on S1 and the first half of S2 (S3) of the cell-adapted strain and that 803L and 976H of the S2 subunit were critical for the cell adaption of this virus (18). The results collectively indicate that mutations in the spike gene and its corresponding amino acids are the basic changes driving the adaptive process, but it is still unknown which mutations are critical in this process. In the present study, by rescuing recombinant PEDVs carrying mosaic spike proteins composed of spike domains derived from Vero cell-adapted attenuated PEDV strain DR13 (DR13^att^) and its parental virus (DR13^par^), which does not replicate efficiently and cause CPE in Vero cells, and by comparing the growth characteristics of these chimeric viruses, we found that substitutions of three amino acids at positions 605, 633, and 891 of the spike protein give rise to the cell-adapted phenotype of the rescued viruses. This study provides a molecular illustration of the cell adaptation of PEDV.

## RESULTS

### The spike gene determines the adaptation of PEDV DR13^att^ to Vero cells.

In order to investigate whether PEDV DR13^att^ acquired its ability to infect Vero cells as a result of adaptive mutations in the S gene, the transfer vector p-PEDV-S^par^-ΔORF3/RLuc was constructed by cloning the S gene of PEDV DR13^par^ (S^par^) into the original transfer vector p-PEDV-S^att^-ΔORF3/RLuc, a key component of the RNA-targeted PEDV reverse genetic system (19). Next, by using the runoff RNA transcripts synthesized from the two linearized transfer vectors as donor RNAs, the rescue of two recombinant viruses, r-S^att^ and r-S^par^, was attempted to verify their adaptation phenotype on Vero cells.

**FIG 1:**
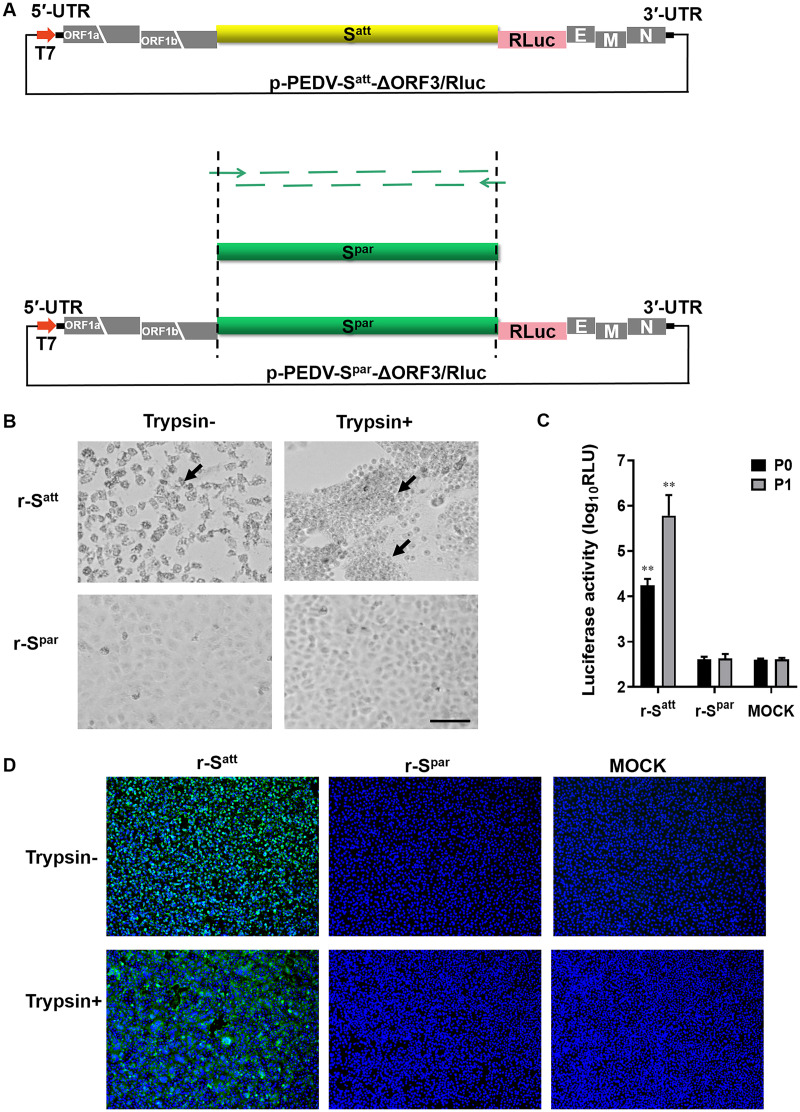
The S gene determines Vero cell adaptation of PEDV DR13^att^. The rescue of recombinant viruses (r-S^par^ and r-S^att^) was carried out by using a reverse genetic system based on homologous RNA recombination. Briefly, LR7 cells infected with mPEDV (a recombinant PEDV with the DR13^att^ backbone carrying the ectodomain of the S gene of mouse hepatitis coronavirus, thereby enabling the virus to infect murine LR7 cells) were harvested. Next, capped runoff RNA transcripts (donor RNA) synthesized from the PacI-linearized transfer vectors were transfected into the mPEDV-infected LR7 cells by electroporation. Finally, the electroporated cells were overlaid onto confluent Vero cells and cultured in the presence or absence of trypsin for the rescue of recombinant viruses, and candidate recombinant viruses were purified by two rounds of endpoint dilutions. (A) Construction of the transfer vector p-PEDV-S^par^-ΔORF3/RLuc. The transfer vector contains the truncated 1a/1b and the structural genes of the PEDV genome. The thick red arrow indicates the T7 promoter from which donor RNA was synthesized *in vitro* using T7 RNA polymerase. The S gene of the PEDV DR13 parental strain (S^par^) (GenBank accession number DQ862099) was obtained by using fusion PCR with artificially synthesized DNA fragments. p-PEDV-S^par^-ΔORF3/RLuc was constructed by replacing the S gene of the PEDV DR13 attenuated strain (S^att^) in p-PEDV-S^att^-ΔORF3/RLuc with the S^par^ gene. UTR, untranslated region. (B) CPE of r-S^par^ and r-S^att^ on Vero cells. Vero cells in 24-well plates were infected with r-S^par^ (its culture) and r-S^att^ in the presence or absence of trypsin (15 μg/mL). The formation of CPE was observed at 36 h postinoculation (hpi). Arrows indicate CPE. Bar, 100 μm. (C) Luciferase expression by r-S^par^ and r-S^att^. The intracellular *Renilla* luciferase activities of the first two generations (P0 and P1) of recombinant viruses were determined when obvious CPE appeared or at 5 days postinoculation (*y* axis) (relative light units [RLU]). The results are expressed as the mean values from three parallel tests, and error bars represent the standard deviations (SD). Comparisons were carried out between the luciferase activities of the recombinant viruses and that of the mock treatments. *, *P < *0.05; **, *P < *0.01. (D) Indirect immunofluorescence assay (IFA) of r-S^par^- and r-S^att^-infected cells. Vero cells were infected with r-S^att^ and r-S^par^ in the presence or absence of trypsin (15 μg/mL). Infected cells were fixed at 36 hpi and immunolabeled with rabbit anti-PEDV M polyclonal antibody (green). Nuclei were labeled with DAPI (blue).

The full-length S^par^ gene (GenBank accession number DQ862099) was synthesized by fusion PCR with 60-nt oligonucleotide fragments (Fig. 1A). By replacing the S^att^ gene (S gene of DR13^att^) with the S^par^ gene, the transfer vector p-PEDV-S^par^-ΔORF3/RLuc was obtained (Fig. 1A). Consistent with our previous results (19), the recombinant virus r-S^att^ was successfully rescued, as evidenced by cell morphology observations, *Renilla* luciferase (RLUC) activity assays, and immunofluorescence assays (IFAs). The CPE was typical of DR13^att^: cells ruptured and detached in the absence of trypsin, or syncytia formed in the presence of trypsin (Fig. 1B). The RLUC values of the first two generations of r-S^att^ were significantly higher than those of the mock treatment (*P < *0.01) (≥4 log_10_ relative light units [RLU] ) (Fig. 1C). IFAs with anti-M monoclonal antibody (mAb) also showed that Vero cells infected with r-S^att^ could form typical CPE in the presence of trypsin (Fig. 1D). However, three rounds of virus rescue with p-PEDV-S^par^-ΔORF3/RLuc did not produce the recombinant virus r-S^par^ in either the presence or the absence of trypsin. The passage of the r-S^par^ culture supernatant did not produce obvious CPE, and the RLUC value remained at a negative level comparable to that of the mock treatment (<4 log_10_ RLU) (Fig. 1C). Also, no virus structural protein could be detected in the Vero cells by IFAs (Fig. 1D). Reverse transcription-PCR (RT-PCR) was also performed with medium or cells from passage 2 (P2) to P4, and no relevant sequences were detected (data not shown). All of these results proved that the recombinant virus carrying S^par^ could not be rescued, while the recombinant virus with the same backbone genome but carrying the S^att^ gene could be successfully generated and propagated, indicating that the S gene alone determines DR13^att^ adaptation to Vero cells.

### PEDV DR13^att^ adaptation to Vero cells maps to the domain from residues 530 to 936 of the S^att^ protein.

Having confirmed that the S gene was responsible for DR13^att^ cell adaptation, we further investigated the main region of S^att^ that conferred this characteristic. To this end, we rescued a series of recombinant viruses carrying a chimeric S gene of S^att^ and S^par^. The survival and growth characteristics of the recombinant viruses were evaluated to define the region of the S gene that determined the cell adaptation of DR13^att^.

**FIG 2:**
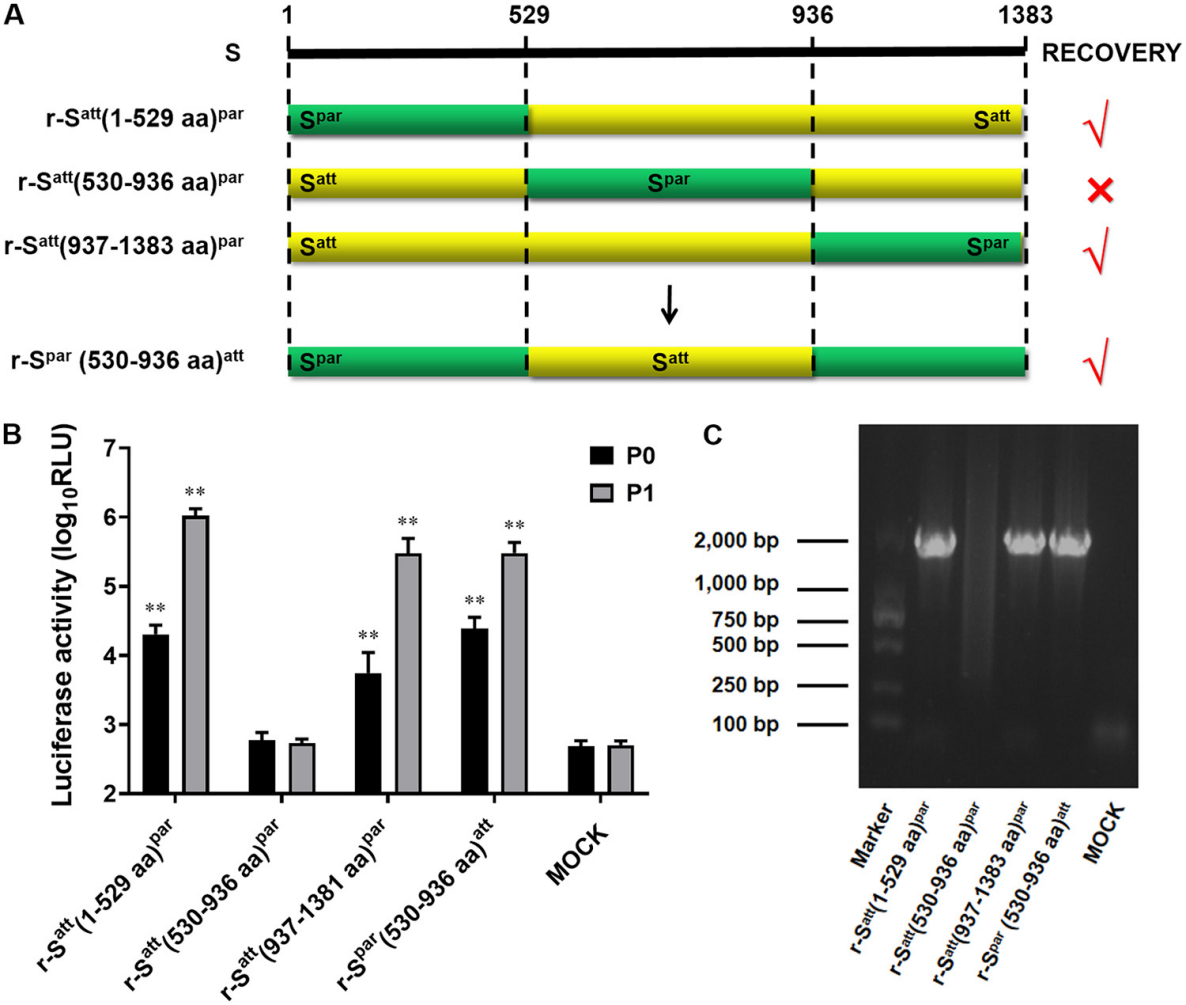
The region of S^att^ from aa 530 to 590 contains a critical residue(s) determining the Vero cell adaptation of PEDV DR13^att^. (A) Schematic representation of the chimeric S proteins of the recombinant viruses r-S^att^(1–529 aa)^par^, r-S^att^(530–936 aa)^par^, r-S^att^(937–1383 aa)^par^, and r-S^par^(530–936 aa)^att^. (B) Luciferase expression by r-S^att^(1–529 aa)^par^, r-S^att^(530–936 aa)^par^, r-S^att^(937–1383 aa)^par^, and r-S^par^(530–936 aa)^att^. The intracellular *Renilla* luciferase activities of the first two generations (P0 and P1) of recombinant viruses were determined (*y* axis) (relative light units [RLU]). The results are expressed as the mean values from three parallel tests, and error bars represent the SD. Comparisons were made between the luciferase activities of the recombinant viruses and those of the mock treatments. *, *P < *0.05; **, *P < *0.01. (C) RT-PCR confirmation of the rescued recombinant PEDVs. RT-PCR was performed covering the region of the S protein from aa 530 to 936 (Primers Ped136 [5′-TGCATCTCGGTTTGTTGGATGC-3′] and Ped137 [5′-TATATTACCAATAGCAGAGTTA-3′]) using the RNA template isolated from the recombinant PEDVs, and the protein was analyzed by gel electrophoresis. The expected size of the RT-PCR product was 1,789 bp.

Supposing that the region from aa 249 to 529 is involved in receptor binding and is likely to be the key region for DR13^att^ cell adaptation, the full-length S protein was initially divided into three segments, aa 1 to 529, aa 530 to 936, and aa 936 to 1381, for a domain-swapping experiment between S^att^ and S^par^ (Fig. 2A). This is a loss-of-function test for virus adaptation to cells: if the replaced segment of S^att^ fails to produce a robust recombinant virus, the replaced segment is expected to be important for adaptation. The recombinant viruses r-S^att^(1–529 aa)^par^ and r-S^att^(937–1381 aa)^par^ were successfully rescued. Vero cell CPE was produced with the two viruses, and the RLUC values of the two recombinant viruses were significantly higher than that of the mock treatment (≥4 log_10_ RLU) (*P < *0.01) (Fig. 2B). RT-PCR of the S genes and sequencing analysis of the two recombinant viruses provided confirmatory results (Fig. 2C). However, the recombinant virus r-S^att^(530–936 aa)^par^ could not be rescued, even after four rounds of attempts (in the presence or absence of trypsin). CPE observation, RLUC activity detection, and RT-PCR all gave negative results (Fig. 2B and C). These observations indicated that the segment of S^att^ from aa 530 to 936 was important for the cell adaptation of the virus. For further confirmation, the transfer vector carrying the entire ectodomain of the S^par^ gene was modified by replacing the region of S^par^ from aa 530 to 936 with that of S^att^, after which the recombinant virus r-S^par^(530–936 aa)^att^ was rescued successfully (Fig. 2B and C), again confirming that the critical region for the adaptation of DR13^att^ existed within the region of S^att^ from aa 530 to 936.

### Narrowing down the mutations responsible for PEDV DR13^att^ cell adaptation to six residues in the S protein.

**FIG 3:**
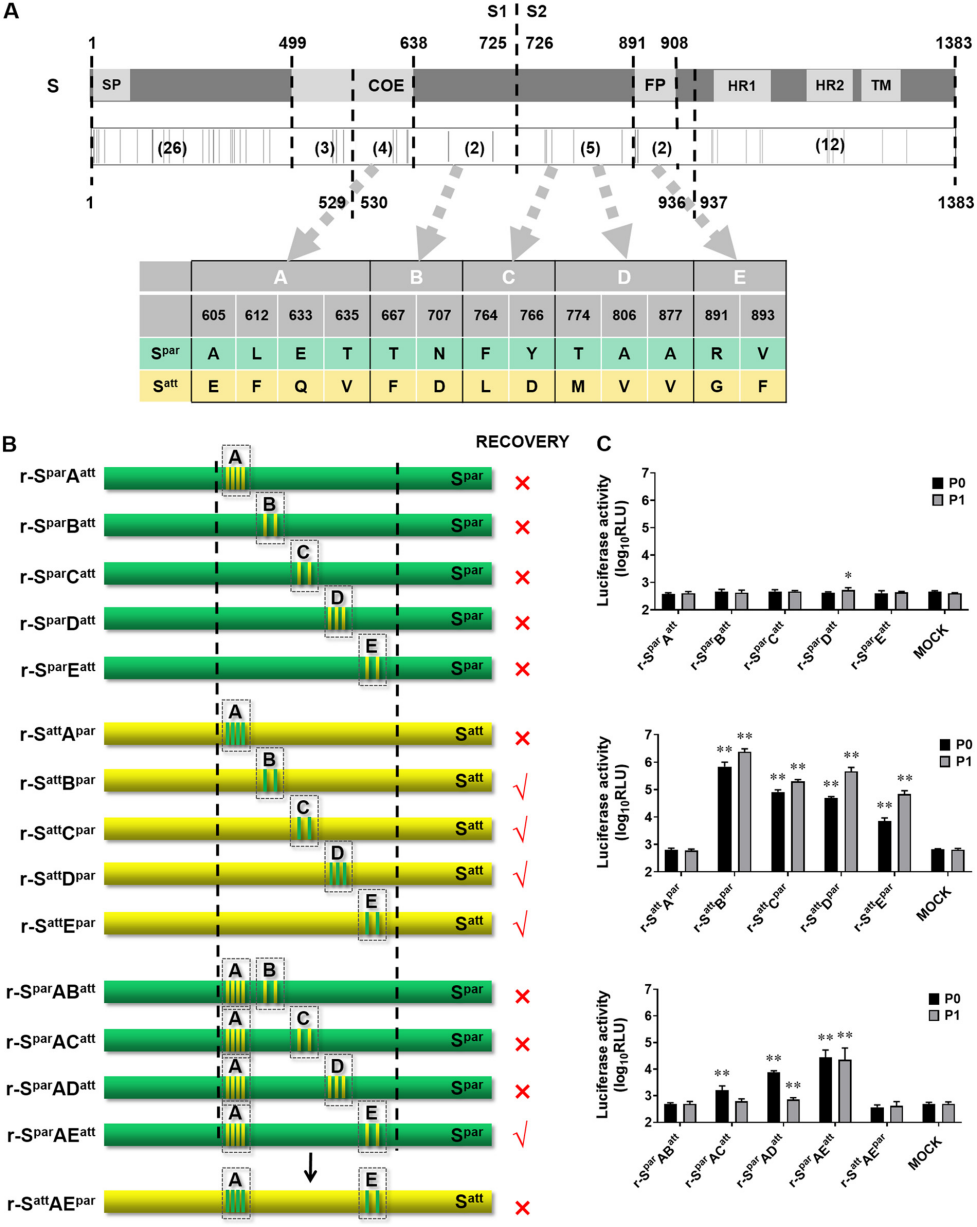
Mapping the exact region determining the Vero cell adaptation of DR13^att^. (A) Schematic representation of the S^par^ protein. the S^par^ protein is symbolically divided into S1 (aa 1 to 725) and S2 (aa 726 to 1383) subunits. The signal peptide (SP) (aa 1 to 18), regions containing a neutralizing epitope (COE) (aa 499 to 638), the fusion peptide (FP) (aa 891 to 908), two heptad repeat regions (HR1 [aa 978 to 1117] and HR2 [aa 1274 to 1313]), and the transmembrane domain (TM) (aa 1324 to 1346) are shown in light gray. The positions of the different amino acids are shown schematically as gray vertical lines below, and the numbers of amino acids differing between S^att^ and S^par^ in the corresponding regions are shown in parentheses. The region of S^att^ and S^par^ from aa 530 to 936 was divided into five groups (A to D), and the amino acid differences are listed in the table below. (B) Schematic representation of the chimeric S proteins. The positions of the mutated residues are shown, and their colors are consistent with the colors of their original S proteins. Dashed boxes refer to the groups defined as described above for panel A. (C) Luciferase expression by recombinant PEDVs with chimeric S genes. The intracellular *Renilla* luciferase activities of the first two generations of the recombinant viruses (P0 and P1) (*y* axis) (relative light units [RLU]) were determined. The results are expressed as the mean values from three parallel tests, and error bars represent the SD. Results of comparisons between the luciferase activities of the recombinant viruses and that of the mock treatments are indicated. *, *P < *0.05; **, *P < *0.01.

To analyze more precisely the region determining the cell adaptation of DR13, the amino acid sequences of the regions from aa 530 to 936 of S^att^ and S^par^ were compared. A total of 13 different amino acids were identified. These 13 variations were further grouped into groups A, B, C, D, and E (Fig. 3A). Among them, group A belongs to the core epitope region, and group E is at the N-terminal end of the fusion peptide (FP). The other groups do not belong to any known functional domain (Fig. 3A). Group mutation tests were carried out one by one in the background of S^par^ or S^att^. Mutations of S^att^-type amino acids introduced into S^par^ are referred to as forward mutations, while mutations of S^par^-type amino acids introduced into S^att^ are referred to as reverse mutations. In this way, 10 different transfer vectors with different chimeric S gene fragments were constructed (Fig. 3B). The rescue experiments showed that none of the five forward group mutations produced viable recombinant virus, as judged by a CPE check and RLUC detection (Fig. 3C). Apparently, the separate introduction of forward group mutations of the S^att^ type to S^par^ could not mediate adaptation to Vero cells. Conversely, four of the five grouped reverse mutations produced viable recombinant viruses; they were named r-S^att^B^par^, r-S^att^C^par^, r-S^att^D^par^, and r-S^att^E^par^, of which r-S^att^E^par^ was weak and could not be checked after passages. The results of CPE observation, RLUC value determination (≥4 log_10_ RLU) (Fig. 3C), as well as RT-PCR analysis supported this conclusion. These results demonstrated that the introduction of the reverse mutation of A^par^ into S^att^ abolished the infectivity of the recombinant virus on Vero cells, while the other grouped reverse mutations did not have this lethal effect, although the introduction of E^par^ affected the viability of the resulting recombinant virus to a considerable extent. These results indicated that the group A mutations in S^att^ were critical for cell adaptation but were not sufficient on their own, probably requiring additional mutations. For the verification of this hypothesis, group A mutations of the S^att^ type were introduced into S^par^ in pairwise combinations with group B to E S^att^-type mutations for constructing transfer vectors (Fig. 3B). The experiments showed that only r-S^par^AE^att^ could be successfully rescued (as shown in Fig. 3B and C). In order to verify the results in an opposite direction, the transfer vector pPEDV-S^att^AE^par^ΔORF3/RLuc was constructed, in which S^par^ type A and E mutations were introduced into S^att^. As expected, no virus was rescued with this transfer vector. It could therefore be concluded that the six mutations in groups A and E in S^att^ were critical for virus adaptation to Vero cells.

### Combined mutations at positions 605, 633, and 891 in S^att^ enable Vero cell infection by DR13^att^.

**FIG 4:**
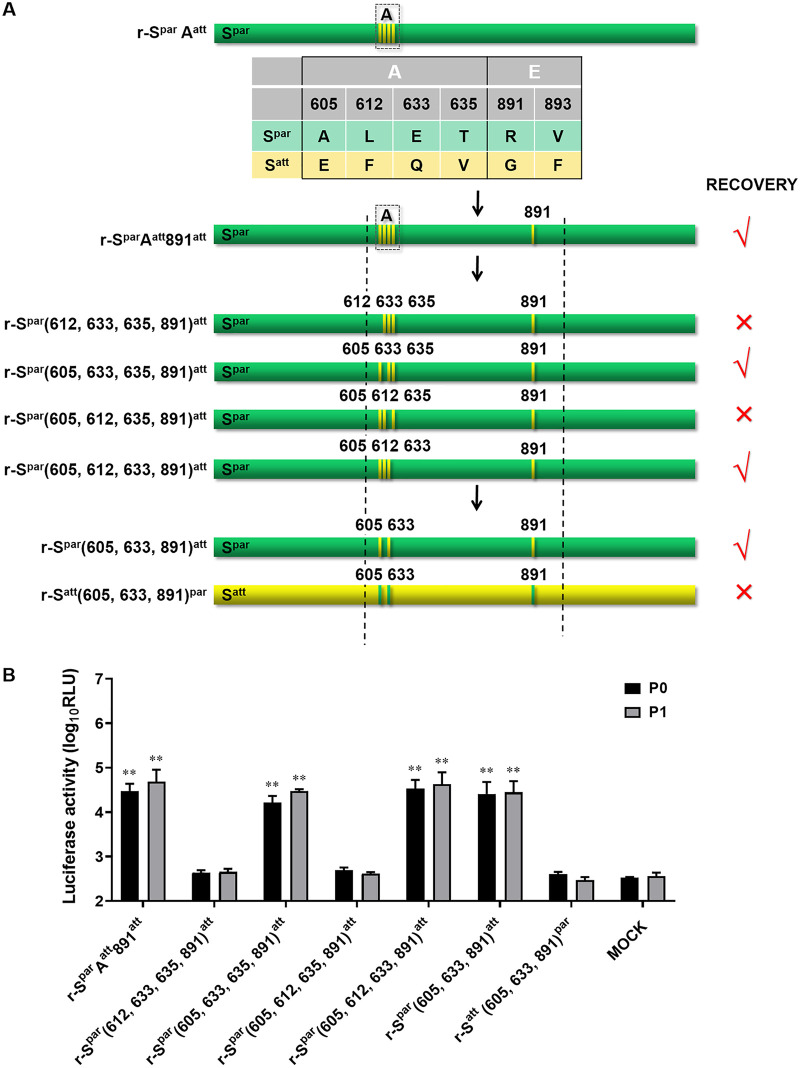
The combination of mutations at positions 603, 633, and 891 of S^att^ largely determines the Vero cell adaptation of DR13^att^. (A) Schematic representation of the chimeric S proteins of recombinant PEDVs. The positions of the mutated residues are shown, and their colors are consistent with the colors of their original S proteins. (B) Luciferase expression by recombinant PEDVs. The intracellular *Renilla* luciferase activities of the first two generations of the recombinant viruses (P0 and P1) (*y* axis) (relative light units [RLU]) were determined. The results are expressed as the mean values from three parallel tests, and error bars represent the SD. Comparisons were carried out between the luciferase activities of the recombinant viruses and that of the mock treatments. *, *P < *0.05; **, *P < *0.01.

To identify the amino acids that play the most critical role in DR13 adaptation to Vero cells among the group A and E mutations, substitutions of the four mutant residues in group A were carried out in combination with the substitution of the amino acid at position 891 in group E (Fig. 4A). First, amino acid R at position 891 of S^par^ was replaced with G in the context of S^par^A^att^, and the recombinant virus, named r-S^par^A^att^891^att^, was rescued. Relative tests confirmed the successful rescue (data not shown). As r-S^par^A^att^ could not be rescued, as described above (Fig. 3B), the 891R→G substitution should be the only variation that makes r-S^par^A^att^891^att^ rescuable. Next, the mutant residues of group A of r-S^par^A^att^891^att^ were replaced one by one with the corresponding amino acids in S^par^ to construct the transfer vectors. The rescue of the intended recombinant viruses r-S^par^(612, 633, 635, 891)^att^ and r-S^par^(605, 612, 635, 891)^att^ was not successful, even with four independent attempts (r-S^par^AE^att^ and the negative control were normal in the four rescue experiments), indicating that replacements of the amino acids with those of the parental type at positions 605 and 633 are not effective (Fig. 4A). In contrast, the intended recombinant viruses r-S^par^(605, 633, 635, 891)^att^ and r-S^par^(605, 612, 633, 891)^att^ were rescued, as confirmed by CPE observation and RLUC values (Fig. 4B). Collectively, these results indicated that three amino acid substitutions at positions 605, 633, and 891 of S^att^ were the minimal mutation combination required for DR13^att^ to adapt to growth in Vero cells.

For further verification of the function of the minimal mutation combination, we constructed two other transfer vectors, pPEDV-S^par^(605, 633, 891)^att^-ΔORF3/RLuc and pPEDV-S^att^(605, 633, 891)^par^-ΔORF3/RLuc (Fig. 4A). The former vector produced viable virus, named r-S^par^(605, 633, 891)^att^ (Fig. 4B). As expected, the latter vector did not produce viable virus. These results demonstrated that the three amino acid mutations at positions 605, 633, and 891 of S^att^ were the minimal mutation composition for the DR13^att^ strain to become adapted to Vero cell cultures.

### Comparison of the adaptation and proliferation characteristics of recombinant viruses in Vero cells.

**FIG 5:**
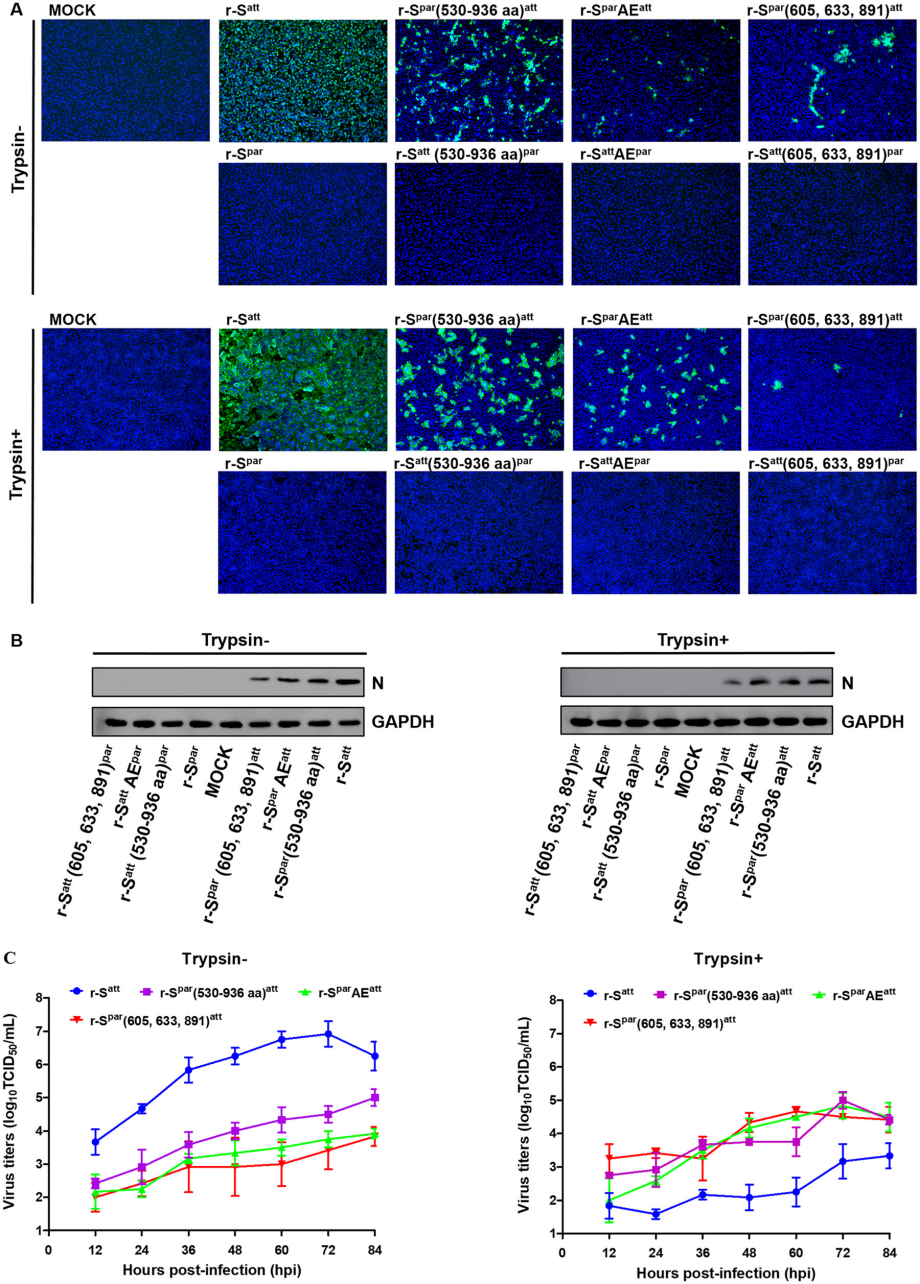
Characterization of the recombinant PEDVs in Vero cells. (A) IFA of recombinant PEDVs. Eight recombinant PEDVs, r-S^par^, r-S^att^, r-S^att^(530–936 aa)^par^, r-S^par^(530–936 aa)^att^, r-S^att^AE^par^, r-S^par^AE^att^, r-S^att^(605, 633, 891)^par^, and r-S^par^(605, 633, 891)^att^, were inoculated into Vero cells in the presence or absence of trypsin (15 μg/mL). Cells were fixed at 36 hpi and immunolabeled with rabbit anti-PEDV M polyclonal antibody (green). Nuclei were labeled with DAPI (blue). (B) Western blot analysis of Vero cells infected with recombinant PEDVs. Vero cells inoculated with recombinant PEDVs were harvested at 24 hpi. Proteins in the lysed cells were separated by 12% SDS-PAGE and subsequently processed for immunoblot analysis with monoclonal antibody against the PEDV N protein; GAPDH served as a protein loading control. (C) Multistep growth kinetics of recombinant PEDVs. Vero cells were inoculated with r-S^att^, r-S^par^(530–936 aa)^att^, r-S^par^AE^att^, and r-S^par^(605, 633, 891)^att^ (MOI of 0.0012) and continued to be cultured in the presence or absence of trypsin (15 μg/mL). At the indicated times postinfection, cells and culture media were harvested by three rounds of freezing and thawing, followed by centrifugation to remove cell debris. The supernatants were collected and used to measure viral titers in the presence of trypsin. Results are expressed as the mean values from three parallel tests, and error bars represent the SD.

In order to compare the characteristics of the rescued viruses under identical conditions, the following rescued viruses were cultured to the eighth passage: r-S^par^, r-S^att^, r-S^att^(530–936 aa)^par^, r-S^par^(530–936 aa)^att^, r-S^att^AE^par^, r-S^par^AE^att^, r-S^att^(605, 633, 891)^par^, and r-S^par^(605, 633, 891)^att^. Vero cells were inoculated with the eighth generation of the viruses in the presence or absence of trypsin, and an IFA was performed with rabbit anti-PEDV M polyclonal antibodies. The results showed that Vero cells inoculated with the rescue cultures of r-S^par^, r-S^att^(530–936 aa)^par^, r-S^att^AE^par^, and r-S^att^(605, 633, 891)^par^ had no obvious CPE and could not be stained by PEDV M polyclonal antibody (Fig. 5A). However, the Vero cells inoculated with the other 4 cultures of r-S^att^, r-S^par^(530–936 aa)^att^, r-S^par^AE^att^, and r-S^par^(605, 633, 891)^att^ could be stained with the M protein antibody. Typical CPE was observed: single-cell rupture without trypsin and syncytia with trypsin. Western blotting with rabbit polyclonal antibody against the PEDV N protein confirmed these results. No corresponding blotting bands were observed with the cells inoculated with the four cultures of r-S^par^, r-S^att^(530–936 aa)^par^, r-S^att^AE^par^, and r-S^att^(605, 633, 891)^par^. The cells inoculated with the other four viruses, r-S^att^, r-S^par^(530–936 aa)^att^, r-S^par^AE^att^, and r-S^par^(605, 633, 891)^att^, showed an obvious protein band with the expected molecular weight of the N protein (53 kDa) (Fig. 5B). Therefore, both IFA and Western blot results reconfirmed that the recombinant viruses r-S^par^, r-S^att^(530–936 aa)^par^, r-S^att^AE^par^, and r-S^att^(605, 633, 891)^par^, all of which had an S protein with amino acids of the parental type at positions 605, 633, and 891, could not be rescued. The same testing protocols reconfirmed the successful rescue of the other four recombinant viruses, r-S^att^, r-S^par^(530–936 aa)^att^, r-S^par^AE^att^, and r-S^par^(605, 633, 891)^att^, all of which had S proteins carrying amino acids of the attenuated type at positions 605, 633, and 891 (Fig. 5B).

To further assess the impact of these key mutations on DR13 adaptation to Vero cells, the four rescued recombinant viruses were inoculated onto Vero cells at the same multiplicity of infection (MOI), and their multistep growth kinetics were analyzed. There was a significant difference in the proliferation rates of the four viruses in the absence of trypsin, which, from high to low, can be arranged in the order r-S^att^, r-S^par^(530–936 aa)^att^, r-S^par^AE^att^, and r-S^par^(605, 633, 891)^att^ (Fig. 5C). The maximal titers of the four viruses ranged from 10^3.8^ to 10^6.9^ 50% tissue culture infective doses (TCID_50_)/mL. The titers of r-S^att^ were significantly higher than those of the other 3 recombinant viruses at all measurement time points, and from 60 h postinoculation (hpi), the titers of r-S^par^(530–936 aa)^att^ were significantly higher than those of r-S^par^AE^att^ and r-S^par^(605, 633, 891)^att^ (*P < *0.05) (Table 1). Trypsin supplementation significantly reduced the proliferation of r-S^att^, with its titer reaching only about 10^3.2^ TCID_50_/mL (Fig. 5C). Upon the addition of trypsin, the titers of r-S^att^ were significantly lower than those of the other 3 recombinant viruses (*P < *0.05) (Table 1). In contrast, trypsin was beneficial for the proliferation of r-S^par^AE^att^ and r-S^par^(605, 633, 891)^att^; their titers increased by nearly an order of magnitude in the presence of trypsin. However, trypsin had no obvious effect on the proliferation of r-S^par^(530–936 aa)^att^. These results indicated that the amino acids at positions 605, 633, and 891 were crucial for the cell adaptation of DR13^att^ but that other amino acid mutations in groups A to E also contributed to a lesser extent. In addition, a significant effect of trypsin on the proliferation of the recombinant viruses was observed.

**TABLE 1:** Significance analysis of the titer differences of recombinant PEDVs at different time points^*a*^

Strain	Mean titer (log_10_ TCID_50_/mL) at hpi						
	12	24	36	48	60	72	84
With trypsin							
r-S^att^	1.83 C	1.58 C	2.16 B	2.08 C	2.25 C	3.17 B	3.33 B
r-S^par^(530–936 aa)^att^	2.75 AB	2.92 AB	3.67 A	3.75 B	3.75 B	5.00 A	4.42 A
r-S^par^AE^att^	2.00 BC	2.58 B	3.50 A	4.17 AB	4.50 A	4.83 A	4.50 A
r-S^par^(605, 633, 891)^att^	3.25 A	3.42 A	3.25 A	4.33 A	4.67 A	4.5 A	4.42 A
Without trypsin							
r-S^att^	3.67 A	4.67 A	5.83 A	6.25 A	6.75 A	6.92 A	6.25 A
r-S^par^(530–936 aa)^att^	2.42 B	2.92 B	3.58 B	4.00 B	4.33 B	4.50 B	5.00 B
r-S^par^AE^att^	2.17 B	2.25 C	3.17 B	3.33 BC	3.50 C	3.75 C	3.92 C
r-S^par^(605, 633, 891)^att^	2.00 B	2.42 BC	2.92 B	2.92 C	3.00 C	3.42 C	3.83 C

a Titers are expressed as the mean values (log_10_ TCID_50_ per milliliter) from three parallel tests. Values marked with the same letter (A, B, or C) are not significantly different (*P* > 0.05); otherwise, the difference is significant (*P* < 0.05).

## DISCUSSION

The isolation of PEDV from clinical samples and subsequent propagation in cultured cells are known to be difficult (12). The virus needs to adapt to the cells, but the precise mechanisms and requirements for this are still not known. Attenuated PEDV DR13 (DR13^att^) is special in that it propagates in cultured cells efficiently independent of trypsin, which is usually needed by other cell-adapted strains for growth *in vitro*, e.g., for strains CV777 and 83P-5. The parental strain, DR13^par^, could not be propagated in cultured cells initially (20). The features of cell adaptation and trypsin independence were endowed to strain DR13^att^ through about 100 passages in Vero cells (9), which was accompanied by 54 amino acid mutations in the S protein. As the S protein of coronaviruses is responsible for virus tropism, infection, and cell adaption (13, 17, 18), we focused this study on the identification of key amino acid mutations in the protein that lead to the Vero cell adaptation of DR13^att^ and the exploration of the underlying mechanism.

First, we verified by swapping the ectodomains of the S proteins of DR13^par^ and DR13^att^ in the recombinant viruses that it was indeed the S protein that determined the adaptation of DR13^att^ (Fig. 1). Next, by constructing a series of recombinant PEDVs carrying chimeric S genes of strains DR13^par^ and DR13^att^, the fragments of the S^att^ protein responsible for virus adaptation to cells and trypsin independence were screened and evaluated. We found that the main part of the S protein responsible for the adapted phenotype was the section comprising residues 530 to 936, which spans the C-terminal part of S1 and the N-terminal domain of S2, nearly half and half, containing key functional domains of the receptor binding domain (RBD), core neutralizing epitope (COE), the S2′ cleavage site, and the fusion peptide (FP). Within this section, we found the combination of the S^att^-type amino acids between positions 605 and 635 (in group A) and one or two of the S^att^-type amino acids at positions 891 and 893 (in group E) to be crucial for the cell adaptation features of the virus (Fig. 3). By exchanging amino acids at these positions and rescuing more recombinant viruses, we demonstrated the composition of the three S^att^-type amino acids at positions 605 and 633 (in group A) and position 891 (in group E) to be sufficient for the adaptation phenotype (Fig. 4). Interestingly, when analyzing the two amino acid differences in group E, we found that the V/F mutation at position 893 and the R/G mutation at position 891 had similar effects; the recombinant virus r-S^par^A^att^893^att^ could also be rescued without trypsin, suggesting that the mutations at positions 893 and 891 play similar roles in endowing the virus with cell adaptation features. Hence, mutations at positions 605, 633, and 891 may not represent the only minimum composition to achieve Vero cell adaptation. Further experiments will be needed to explore other potential minimum compositions of S^att^-type mutations that may support the cell adaptation of PEDV.

While the S^att^-type mutations at positions 605, 633, and 891 are critical for Vero cell adaptation, S^att^-type mutations at other positions additionally promoted virus infection. This can be deduced from the observation that introducing additional mutations at sites of difference between the parental and adapted DR13 virus S proteins facilitated the rescue of the recombinant viruses and gave rise to the earlier development of CPE and higher viral titers (Fig. 5). Therefore, while mutations at a few sites in the S protein are primarily responsible for adaptation, mutations at additional sites function to further enhance viral competence in the new host. If we examine the location of the three key amino acids in the spike structure, two of them (aa 605 and aa 633) appear to reside in or close to the RBD, which is assumed to interact with the cell receptor (21), while the third residue (aa 891) occurs just prior to or at the N terminus of the FP domain, C terminally to the assumed S2′ site (22). Both the RBD and S2′ are key domains for determining virus tropism through binding to their specific receptor and interacting with organ- or tissue-specific proteases (22, 23).

Our study showed that trypsin supplementation in the culture medium decreased the titers of r-S^att^ but not those of the other rescued viruses that we tested (Fig. 5C), which is somewhat puzzling considering the very few differences in their S proteins. This phenomenon may result from the specific mutation composition of DR13^att^, whose alternative cleavage site becomes more readily accessible to environmental proteases than in the other viruses, thereby triggering the premature transition of the S protein into a postfusion form and leading to a decreased infection ability (24). The special features of the DR13^att^ strain with respect to trypsin independence, the S protein cleavage pattern, and attenuated virulence indicated that DR13^att^ may use a strategy for S protein activation different from that of its parental strain or other wild-type PEDVs, which use trypsin to activate the S protein and R891 as the S2′ cleavage site (22). The R891G/V893F mutation at the N-terminal end of FP could make the mutated S2′ site switch to another protease residing on the cell membrane or in the endosome, which cleaves the S protein during or after virus entry (25). Taking these results together, we hypothesize that DR13^att^ employed a cell entry strategy different from that of its parental generations. On the one side, the mutations at positions 891 and 893 abolished the original trypsin cleavage site and at the same time exposed an alternative site to another protease that assists the virus in entering the cells through endocytosis (26, 27). On the other side, the DR13^att^ strain could have found a new receptor on Vero cells or a new way to interact with the receptor, as could be inferred from the mutations at positions 605 and 633, which may cause an alteration in the three-dimensional (3D) structure of the RBD and a change in the dynamic process of ligand-receptor interactions (23, 28). No doubt, it was the culture conditions of the virus that dominated such a selection, as inconsistent trypsin activity during passaging but the stable physical chemistry environment of the culture system made the selection of an adapted virus possible.

Recently, Li et al. found that the collaboration of S1 and the first half of S2 (S3) of the S protein determined the adaptability of PEDV to Vero cells (18). Their findings conformed to the theoretical assumption that the receptor binding domain as well as the cleavage site determine PEDV’s tropism (21, 22). However, their observation that the 803L and 976H residues of the S3 subunit are critical for the function of the first half of S2 was more or less out of expectation considering that these two sites do not comprise cleavage sites or the fusion peptide. Those authors hypothesized that S2 involved S1 binding to the receptor or involved membrane adsorption mediated by FP, but how residues 803L and 976H played their role in these processes is still a conundrum. From the perspective of our research, the mutations, whether related to the RBD or the cleavage site of the S protein, could produce an effect on virus tropism or protease selection, but different strains may use different strategies, as expressed by different mutation compositions in the S genes, betokening the underlying elusive mechanism.

In conclusion, by using the RNA-targeted reverse genetic system of PEDV, S protein swapping, and recombinant virus rescue, we identified three key amino acid mutations in the S protein of PEDV DR13^att^, A605E, E633Q, and R891G, that are mainly responsible for the Vero cell adaptation of the strain. The former two key mutations reside inside and in the vicinity of the RBD, respectively, while the latter one is located at the N-terminal end of FP. Besides the three key mutations, other mutations in the S protein conferred additional viability to the viruses. We hypothesize that the three mutations changed the virus’s tropism by changing the S2′ cleavage site and its RBD-receptor interaction. These findings provide a reference for further mechanistic investigations into PEDV cell adaptation and PEDV vaccine design.

## MATERIALS AND METHODS

### Cells, viruses, and transfer vectors.

Murine L (LR7) cells (an L-2 murine fibroblast cell line stably expressing the murine hepatitis virus receptor; a gift from Peter J. M. Rottier, Utrecht University) and Vero ATCC CCL-81 cells (African green monkey kidney cells; purchased from the ATCC) were cultured in Dulbecco’s modified Eagle’s medium (DMEM; Gibco, Invitrogen, Carlsbad, CA, USA) supplemented with 10% fetal bovine serum (FBS; Gibco, Invitrogen, Carlsbad, CA, USA), penicillin (100 U/mL), and streptomycin (100 μg/mL) at 37°C in a humidified atmosphere with 5% CO_2_.

mPEDV, a recombinant PEDV carrying the ectodomain of the S protein of mouse hepatitis coronavirus, thereby enabling the virus to infect murine LR7 cells, and the transfer vector p-PEDV-S^att^-ΔORF3/RLuc were prepared as described in our previous study (16). The cell culture-adapted DR13^att^ strain (GenBank accession number JQ023162) (isolated from a commercial vaccine from GreenCross, South Korea) and the rPEDVs were propagated and titrated in Vero cells.

### Synthesis of the DR13 S^par^ gene and construction of the p-PEDV-S^par^-ΔORF3/RLuc transfer vector.

According to the S gene sequence of the PEDV DR13 parent strain (DR13^par^) provided by the NCBI (GenBank accession number DQ862099), a total of 108 primers of 60 nucleotides (nt) overlapping each other by 21 nt were designed and artificially synthesized (Sangon Biotech, Shanghai, China), from which the complete S gene of DR13^par^ (S^par^) was synthesized by fusion PCR. Both ends of the amplified fragment 1b′-S^par^-RLuc′ had an overhanging 42-nucleotide-long homologous fragment of the transfer vector. The vector fragment PEDV′-ΔS^att^-ΔORF3/RLuc′ (without the S^att^ gene) was synthesized by PCR with the vector p-PEDV-S^att^-ΔORF3/RLuc as the template using primer pair Ped138/Ped139 (Table 2). The 1b′-S^par^-RLuc′ fragment was cloned into the PEDV′-ΔS^att^-ΔORF3/RLuc′ vector using a homologous recombination method (ClonExpress MultiS one-step cloning kit; Vazyme, Nanjing, China) to construct the transfer vector p-PEDV-S^par^-ΔORF3/RLuc. The acquired recombinant plasmid was verified by sequencing.

**TABLE 2:** Primers for constructing transfer vectors used to rescue recombinant PEDVs containing chimeric S genes

Recombinant virus	Transfer vector	Amplified fragment^*a*^	Template	Primer name	Primer location (positions)^*b*^	Primer sense	Primer sequence (5′–3′)
r-S^par^	p-PEDV-S^par^-ΔORF3/RLuc	PEDV′-ΔS^att^-ΔORF3/RLuc′	p-PEDV-S^att^-ΔORF3/RLuc	Ped138	−42 to −1	−	TTGTTTACGTTGACCAAATGATTAGAAAAGCCACAAATGGCG
				Ped139	End +1 to end +42	+	GCTAGCCACCATGACTTCGAAAGTTTATGATCCAGAACAAAG
		1b′-S^par^-RLuc′	1b′-S^par^-RLuc′	Ped140	−42 to −1	+	CGCCATTTGTGGCTTTTCTAATCATTTGGTCAACGTAAACAA
				Ped141	End +1 to end +42	−	CTTTGTTCTGGATCATAAACTTTCGAAGTCATGGTGGCTAGC
							
r-S^att^(1–529 aa)^par^	p-PEDV-S^att^(1–529 aa)^par^-ΔORF3/RLuc	PEDV′-ΔS^att^-ΔORF3/RLuc′	p-PEDV-S^att^-ΔORF3/RLuc	Ped138	−42 to −1	−	TTGTTTACGTTGACCAAATGATTAGAAAAGCCACAAATGGCG
				Ped139	End +1 to end +42	+	GCTAGCCACCATGACTTCGAAAGTTTATGATCCAGAACAAAG
		1b′-(1–529 aa)^par^′	1b′-S^par^-RLuc′	Ped140	−42 to −1	+	CGCCATTTGTGGCTTTTCTAATCATTTGGTCAACGTAAACAA
				Ped151	1598 to 1620	−	ACAGAAAGAACTAAACCCATTGA
		(530–1381 aa)^att^′-RLuc′	p-PEDV-S^att^-ΔORF3/RLuc	Ped146	1586 to 1610	+	CTGACACTACTATCAATGGGTTTAG
				Ped141	End +1 to end +42	−	CTTTGTTCTGGATCATAAACTTTCGAAGTCATGGTGGCTAGC

r-S^att^(530–936 aa)^par^	p-PEDV-S^att^(530–936 aa)^par^-ΔORF3/RLuc	PEDV′-ΔS^att^-ΔORF3/RLuc′	p-PEDV-S^att^-ΔORF3/RLuc	Ped138	−42 to −1	−	TTGTTTACGTTGACCAAATGATTAGAAAAGCCACAAATGGCG
				Ped139	End +1 to end +42	+	GCTAGCCACCATGACTTCGAAAGTTTATGATCCAGAACAAAG
		1b′-(1–529 aa)^att^′	p-PEDV-S^att^-ΔORF3/RLuc	Ped140	−42 to −1	+	CGCCATTTGTGGCTTTTCTAATCATTTGGTCAACGTAAACAA
				Ped151	1598 to 1620	−	ACAGAAAGAACTAAACCCATTGA
		(530–936 aa)^par^′	1b′-S^par^-RLuc′	Ped146	1586 to 1610	+	CTGACACTACTATCAATGGGTTTAG
				Ped147	2786 to 2810	−	ACCATGACACCAGAGTAATACTGCG
		(936–1381 aa)^att^′-RLuc′	p-PEDV-S^att^-ΔORF3/RLuc	Ped154	2786 to 2810	+	CGCAGTATTACTCTGGTGTCATGGT
				Ped141	End +1 to end +42	−	CTTTGTTCTGGATCATAAACTTTCGAAGTCATGGTGGCTAGC

r-S^att^(936–1381 aa)^par^	p-PEDV-S^att^(937–1381 aa)^par^-ΔORF3/RLuc	PEDV′-ΔS^att^-ΔORF3/RLuc′	p-PEDV-S^att^-ΔORF3/RLuc	Ped138	−42 to −1	−	TTGTTTACGTTGACCAAATGATTAGAAAAGCCACAAATGGCG
				Ped139	End +1 to end +42	+	GCTAGCCACCATGACTTCGAAAGTTTATGATCCAGAACAAAG
		1b′-(1–936 aa)^att^′	p-PEDV-S^att^-ΔORF3/RLuc	Ped140	−42 to −1	+	CGCCATTTGTGGCTTTTCTAATCATTTGGTCAACGTAAACAA
				Ped147	2786 to 2810	−	ACCATGACACCAGAGTAATACTGCG
		(530–1381 aa)^par^′-RLuc′	1b′-S^par^-RLuc′	Ped154	2786 to 2810	+	CGCAGTATTACTCTGGTGTCATGGT
				Ped141	End +1 to end +42	−	CTTTGTTCTGGATCATAAACTTTCGAAGTCATGGTGGCTAGC

r-S^par^(530–936 aa)^att^	p-PEDV-S^par^(530–936 aa)^att^-ΔORF3/RLuc	PEDV′-ΔS^att^-ΔORF3/RLuc′	1b′-S^par^-RLuc′	Ped138	−42 to −1	−	TTGTTTACGTTGACCAAATGATTAGAAAAGCCACAAATGGCG
				Ped139	End +1 to end +42	+	GCTAGCCACCATGACTTCGAAAGTTTATGATCCAGAACAAAG
		1b′-(1–529 aa)^par^′	1b′-S^par^-RLuc′	Ped140	−42 to −1	+	CGCCATTTGTGGCTTTTCTAATCATTTGGTCAACGTAAACAA
				Ped151	1598 to 1620	−	ACAGAAAGAACTAAACCCATTGA
		(530–936 aa)^att^′	p-PEDV-S^att^-ΔORF3/RLuc	Ped146	1586 to 1610	+	CTGACACTACTATCAATGGGTTTAG
				Ped147	2786 to 2810	−	ACCATGACACCAGAGTAATACTGCG
		(936–1381 aa)^par^′-RLuc′	1b′-S^par^-RLuc′	Ped154	2786 to 2810	+	CGCAGTATTACTCTGGTGTCATGGT
				Ped141	End +1 to end +42	−	CTTTGTTCTGGATCATAAACTTTCGAAGTCATGGTGGCTAGC

a “′” indicates that the DNA fragment contains homologous arm sequences (flanking sequences) required for subsequent homologous recombination.

b The location of the primers is relative to the S gene sequence of the PEDV DR13^par^ strain (GenBank accession number DQ862099).

### Construction of transfer vectors carrying the chimeric S gene of S^att^ and S^par^.

The chimeric S genes and their transfer vectors were synthesized or constructed with the same homologous recombination method as the one described above. Usually, the shorter S gene segments, for instance, S^par^ aa 1 to 529, aa 530 to 936, and aa 937 to 1381, used for substitution were synthesized by PCR with overhanging short sequences homologous to the longer S gene segments to which they would be fused. The synthesis of these vectors as well as the gene segments was carried out by PCR with the vector p-PEDV-S^par^-ΔORF3/RLuc or p-PEDV-S^att^-ΔORF3/RLuc as the template. The method of point mutation (QuikChange Lightning Multi site-directed mutagenesis kit; Agilent, Palo Alto, CA, USA) was also extensively used for vector constructs that needed only one or a few point mutations in the corresponding vectors (Table 3). The construction of the transfer vectors was confirmed by sequencing.

**TABLE 3:** Point mutation primers for constructing transfer vectors used to rescue recombinant PEDVs

Recombinant virus	Transfer vector	Point mutation template	Primer name	Primer location (positions)^*a*^	Primer sequence (5′–3′)^*b*^	Mutant amino acid site(s)
r-S^par^A^att^	p-PEDV-S^par^A^att^-ΔORF3/RLuc	p-PEDV-S^par^-ΔORF3/RLuc	PEDmutant1	1807–1840	TACCCTG**A**GTTCGGTAGTGGTGTTAAGTT**T**ACGT	605, 612
			PEDmutant2	1881–1907	CACGCCTAAACCACTT**C**AAGGT**G**TCAC	633, 635
						
r-S^par^B^att^	p-PEDV-S^par^B^att^-ΔORF3/RLuc	p-PEDV-S^par^-ΔORF3/RLuc	PEDmutant3	1984–2008	CTTACAAATTCTAGC**T**TTTTGGCAG	667
			PEDmutant4	2101–2126	GAGCAGGCTGCATATGTT**G**ATGATG	707

r-S^par^C^att^	p-PEDV-S^par^C^att^-ΔORF3/RLuc	p-PEDV-S^par^-ΔORF3/RLuc	PEDmutant5	2278–2302	GGCTATGTTCCAC**T**TCAG**G**ATGGCC	764, 766

r-S^par^D^att^	p-PEDV-S^par^D^att^-ΔORF3/RLuc	p-PEDV-S^par^-ΔORF3/RLuc	PEDmutant6	2306–2334	GTCAAGATTGCACCCA**T**GGTTACTGGG	774
			PEDmutant7	2407–2429	GTTGATTGTG**T**TACATATGTTTG	806
			PEDmutant8	2616–2637	CTAATGTGCTGGGTG**T**TTCCGTG	877

r-S^par^E^att^	p-PEDV-S^par^E^att^-ΔORF3/RLuc	p-PEDV-S^par^-ΔORF3/RLuc	PEDmutant9	2662–2688	TGGTACAAAAA**G**GGTCT**T**TTATTGAAG	891, 893
r-S^att^A^par^	p-PEDV-S^att^A^par^-ΔORF3/RLuc	p-PEDV-S^att^-ΔORF3/RLuc	PEDmutant11	1807–1840	TACCCTG**C**GTTCGGTAGTGGTGTTAAGTT**G**ACGT	605, 612
			PEDmutant12	1881–1907	CACGCCTAAACCACTT**G**AAGGT**A**TCAC	633, 635

r-S^att^B^par^	p-PEDV-S^att^B^par^-ΔORF3/RLuc	p-PEDV-S^att^-ΔORF3/RLuc	PEDmutant13	1984–2008	CTTACAAATTCTAGC**A**TTTTGGCAG	667
			PEDmutant14	2101–2126	GAGCAGGCTGCATATGTT**A**ATGATG	707

r-S^att^C^par^	p-PEDV-S^att^C^par^-ΔORF3/RLuc	p-PEDV-S^att^-ΔORF3/RLuc	PEDmutant15	2278–2302	GGCTATGTTCCAC**C**TCAG**T**ATGGCC	764, 766

r-S^att^D^par^	p-PEDV-S^att^D^par^-ΔORF3/RLuc	p-PEDV-S^att^-ΔORF3/RLuc	PEDmutant16	2306–2334	GTCAAGATTGCACCCA**C**GGTTACTGGG	774
			PEDmutant17	2407–2429	GTTGATTGTG**C**TACATATGTTTG	806
			PEDmutant18	2616–2637	CTAATGTGCTGGGTG**C**TTCCGTG	877

r-S^att^E^par^	p-PEDV-S^att^E^par^-ΔORF3/RLuc	p-PEDV-S^att^-ΔORF3/RLuc	PEDmutant19	2660–2688	TGGTACAAAAA**A**GGTCT**G**TTATTGAAG	891, 893

r-S^par^AB^att^	p-PEDV-S^par^AB^att^-ΔORF3/RLuc	p-PEDV-S^par^A^att^-ΔORF3/RLuc	PEDmutant3	1984–2008	CTTACAAATTCTAGC**T**TTTTGGCAG	667
			PEDmutant4	2101–2126	GAGCAGGCTGCATATGTT**G**ATGATG	707

r-S^par^AC^att^	p-PEDV-S^par^AC^att^-ΔORF3/RLuc	p-PEDV-S^par^A^att^-ΔORF3/RLuc	PEDmutant5	2278–2302	GGCTATGTTCCAC**T**TCAG**G**ATGGCC	764, 766

r-S^par^AD^att^	p-PEDV-S^par^AD^att^-ΔORF3/RLuc	p-PEDV-S^par^A^att^-ΔORF3/RLuc	PEDmutant6	2306–2334	GTCAAGATTGCACCCA**T**GGTTACTGGG	774
			PEDmutant7	2407–2429	GTTGATTGTG**T**TACATATGTTTG	806
			PEDmutant8	2616–2637	CTAATGTGCTGGGTG**T**TTCCGTG	877

r-S^par^AE^att^	p-PEDV-S^par^AE^att^-ΔORF3/RLuc	p-PEDV-S^par^A^att^-ΔORF3/RLuc	PEDmutant9	2660–2688	TGGTACAAAAA**G**GGTCT**T**TTATTGAAG	891, 893

r-S^att^AE^par^	p-PEDV-S^att^AE^par^-ΔORF3/RLuc	p-PEDV-S^att^A^par^-ΔORF3/RLuc	PEDmutant19	2660–2688	TGGTACAAAAA**A**GGTCT**G**TTATTGAAG	891, 893

r-S^par^A^att^891^att^	p-PEDV-S^par^A^att^891^att^-ΔORF3/RLuc	p-PEDV-S^par^A^att^-ΔORF3/RLuc	PEDmutant10	2662–2686	GTACAAAAA**G**GGTCTGTTATTGAAG	891

r-S^par^A^att^893^att^	p-PEDV-S^par^A^att^893^att^-ΔORF3/RLuc	p-PEDV-S^par^A^att^-ΔORF3/RLuc	PEDmutant24	2662–2686	GTACAAAAAGGGTCT**T**TTATTGAAG	893

r-S^par^(612, 633, 635, 891)^att^	p-PEDV-S^par^(612, 633, 635, 891)^att^-ΔORF3/RLuc	p-PEDV-S^par^A^att^891^att^-ΔORF3/RLuc	PEDmutant20	1807–1840	TACCCTG**C**GTTCGGTAGTGGTGTTAAGTTTACGT	605

r-S^par^(605, 633, 635, 891)^att^	p-PEDV-S^par^(605, 633, 635, 891)^att^-ΔORF3/RLuc	p-PEDV-S^par^A^att^891^att^-ΔORF3/RLuc	PEDmutant21	1807–1840	TACCCTGAGTTCGGTAGTGGTGTTAAGTT**G**ACGT	612

r-S^par^(605, 612, 635, 891)^att^	p-PEDV-S^par^(605, 612, 635, 891)^att^-ΔORF3/RLuc	p-PEDV-S^par^A^att^891^att^-ΔORF3/RLuc	PEDmutant22	1881–1907	CACGCCTAAACCACTT**G**AAGGTGTCAC	633

r-S^par^(605, 612, 633, 891)^att^	p-PEDV-S^par^(605, 612, 633, 891)^att^-ΔORF3/RLuc	p-PEDV-S^par^A^att^891^att^-ΔORF3/RLuc	PEDmutant23	1881–1907	CACGCCTAAACCACTTCAAGGT**A**TCAC	635

r-S^par^(605, 633, 891)^att^	p-PEDV-S^par^(605, 633, 891)^att^-ΔORF3/RLuc	p-PEDV-S^par^(605, 633, 635, 891)^att^-ΔORF3/RLuc	PEDmutant23	1881–1907	CACGCCTAAACCACTTCAAGGT**A**TCAC	635

r-S^att^(605, 633, 891)^par^	p-PEDV-S^att^(605, 633, 891)^par^-ΔORF3/RLuc	p-PEDV-S^att^-ΔORF3/RLuc	PEDmutant20	1807–1840	TACCCTG**C**GTTCGGTAGTGGTGTTAAGTTTACGT	605
			PEDmutant22	1881–1907	CACGCCTAAACCACTT**G**AAGGTGTCAC	633
			PEDmutant24	2662–2686	GTACAAAAA**G**GGTCTTTTATTGAAG	891

a The location of the primers is relative to the S gene sequence of the PEDV DR13^par^ strain (GenBank accession number DQ862099).

b The mutated nucleotides of the primers are marked in boldface type.

### Targeted RNA recombination.

The generation and rescue of recombinant PEDVs were performed as described previously (19). Briefly, LR7 cells were grown to 90% confluence and infected with mPEDV at a multiplicity of infection (MOI) of 1.0. When obvious cytopathic effect (CPE) started to appear at around 8 h postinoculation (hpi), cells were treated with trypsin to produce a single-cell suspension and washed three times with phosphate-buffered saline (PBS). Capped runoff RNA transcripts (donor RNA) were synthesized from the PacI-linearized transfer vectors using a T7 RNA polymerase kit (Ambion, Carlsbad, CA, USA). Next, the donor RNAs were transfected into the above-described mPEDV-infected LR7 cells by electroporation (300 V, 975 μF, and two consecutive pulses) with a Gene Pulser apparatus (Scientz-2C; Ningbo Scientz Biotech Inc., Ningbo, China). Finally, the electroporated cells were resuspended in DMEM and overlaid onto confluent Vero cells cultured in a 25-cm^2^ flask. The rescue of the recombinant PEDV with trypsin required the addition of 15 μg/mL of trypsin to the culture system 4 h after the overlay incubation. After 4 to 5 days of incubation at 37°C, progeny viruses in the culture supernatant were harvested by freeze-thawing three times, and candidate recombinant viruses were purified by two rounds of endpoint dilutions on Vero cells. Recombinant genotypes were confirmed by RT-PCR and subsequent sequencing.

### Propagation of recombinant PEDV.

Recombinant viruses were propagated as follows. Vero cells at 90% confluence were washed 3 times with PBS and inoculated with the recombinant virus. After a 2-h incubation, the inoculum was removed, and the cells were cultured for 4 to 5 days. The viruses were harvested by centrifugation of the medium after freezing and thawing the culture cells 3 times. When trypsin needed to be added, Vero cells at 100% confluence were inoculated with recombinant viruses, and the cells were cultured for 4 to 5 days in DMEM supplemented with 15 μg/mL trypsin until harvest.

### *Renilla* luciferase assay.

Vero cell monolayers in a T25 flask were inoculated with 2 mL of the harvested recombinant virus cultures. When obvious CPE appeared or at 5 days postinoculation (dpi), both the culture medium and cells were collected for luciferase activity assays using the *Renilla* luciferase assay kit (Promega, Shanghai, China) according to the manufacturer’s instructions, and the relative light units (RLU) were determined with a Berthold Centro LB 960 plate illuminometer (GloMax-Multi detection system; Promega, Madison, WI, USA).

### Virus titration.

Viral titers were measured in 96-well plates using 50% tissue culture infective dose (TCID_50_) assays. The culture supernatants were serially diluted 10-fold from 10^−1^ to 10^−8^, in triplicate per dilution, and added to a monolayer of Vero cells in 96-well culture plates. At 5 dpi, the virus titers were determined according to the Reed-Muench method (29) and expressed as TCID_50_ per milliliter.

### Indirect immunofluorescence analysis.

Vero cells at 90% confluence in 48-well plates were inoculated with 200 μL of recombinant virus cultures. After a 2-h incubation, the inoculum was removed, and cells were cultured in DMEM with or without trypsin (15 μg/mL). At 36 hpi, the cells were rinsed with PBS three times, fixed with 3.7% formaldehyde for 15 min at room temperature, and permeabilized with 0.25% Triton X-100 in PBS for 15 min at 37°C. Next, the cells were blocked with 5% goat serum at 37°C for 1 h and incubated with rabbit anti-PEDV M polyclonal antibody at a dilution of 1:1,000 at 37°C for 1 h. After three washes with PBS, Alexa Fluor 488-conjugated goat anti-rabbit antibody (Beyotime, Shanghai, China) was added at a dilution of 1:200, and the cells were further incubated for 1 h. Finally, the nuclei were stained with 4′,6-diamidino-2-phenylindole (DAPI) (1:1,000 dilution) at 37°C for 15 min, and the cells were washed three times with PBS. Pictures of immunofluorescent cells were captured using an Evos fluorescence microscope (M7000; Thermo Fisher Scientific, Waltham, MA, USA).

### Western blotting.

Vero cells at 90% confluence in a 10-cm culture dish were inoculated with 2 mL of recombinant virus cultures. After 2 h of incubation, the inoculum was removed, and the cells were cultured for 24 h in DMEM with or without trypsin (15 μg/mL). The cells were harvested and lysed with 200 μL of ice-cold radioimmunoprecipitation assay (RIPA) buffer (TransGen, Beijing, China) for 30 min on ice. The supernatant was collected by centrifugation at 12,000 × *g* for 15 min at 4°C. The proteins in the supernatant were separated by 12% SDS-PAGE and transferred onto a polyvinylidene difluoride (PVDF) membrane (Millipore, Merck KGaA, Darmstadt, Germany). The membrane loaded with the transferred proteins was blocked with 5% nonfat milk at 25°C for 1 h and then incubated with rabbit anti-PEDV N monoclonal antibody (Shanghai Ango, Shanghai, China) at a dilution of 1:1,000 or anti-glyceraldehyde-3-phosphate dehydrogenase (GAPDH) polyclonal antibody (Sangon Biotech, Shanghai, China) at a dilution of 1:10,000 in PBS containing 0.1% Tween 20 (PBST) at 25°C for 1 h. After washing with PBST, the membranes were further incubated with horseradish peroxidase (HRP)-conjugated goat anti-rabbit IgG (Sangon Biotech, Shanghai, China) at a dilution of 1:10,000 at 25°C for 1 h. Proteins were detected by using the Amersham ECL Western blotting analysis system (GE Healthcare, Chicago, IL, USA).

### Viral multistep growth kinetics.

Vero cells at 90% confluence in 6-well plates were inoculated with four recombinant viruses at an MOI of 0.0012 in the presence or absence of trypsin (15 μg/mL). After inoculation at 37°C for 2 h, the inoculum was removed, and the cells were further cultured in DMEM with or without 15 μg/mL trypsin. At 12, 24, 48, 60, 72, and 84 hpi, the cultures were collected and stored in aliquots at −80°C. Virus titrations were performed in triplicate in Vero cells. The virus titers were determined by TCID_50_ assays, with which the growth curves were plotted.

### Statistical analysis.

Numerical data were expressed as means ± standard errors of the means (SEM) and were analyzed using GraphPad Prism software (version 9.4; GraphPad Software Inc.). Differences between groups were analyzed by one-way analysis of variance (ANOVA). SPSS v21.0 software for windows (SPSS Inc., Chicago, IL, USA) was used for statistical analysis. A *P* value of <0.05 was considered statistically significant (indicated by * in the figures), and a *P* value of <0.01 was considered extremely significant (indicated by ** in the figures).
